# Editorial: Addressing the sustainable development goals “leave no one behind” promise: migration and health

**DOI:** 10.3389/fpubh.2023.1192454

**Published:** 2023-05-02

**Authors:** Shanquan Chen, Chi Kin Law, Wai-kit Ming, Stefano Orlando

**Affiliations:** ^1^Faculty of Epidemiology and Population Health, London School of Hygiene & Tropical Medicine, London, United Kingdom; ^2^NHMRC Clinical Trials Centre, Faculty of Medicine and Health, The University of Sydney, Camperdown, NSW, Australia; ^3^Department of Infectious Diseases and Public Health, City University of Hong Kong, Kowloon, Hong Kong SAR, China; ^4^Department of Biomedicine and Prevention, University of Rome Tor Vergata, Rome, Italy

**Keywords:** migration, urban-rural dual division system, people-centered integrated care, medical insurance, internal migrant men who have sex with men, rare diseases, migrant older adults following children

## Introduction

Migration (including internal and international) and health are increasingly recognized as a global public health priority, given that in many countries, equitable access to health services is considered a goal only concerning citizens or local residents. A clear call to the United Nations' Sustainable Development Goals (SDGs) is to “leave no one behind,” regardless of legal status, to achieve Universal Health Coverage (UHC) for all (1). The negative impact of absent or limited action on migration works not only on those who migrate, but also on sending, receiving, and “left-behind” communities. The present Research Topic is a response to the call from the SDGs with an especially focus on the health issues of migrants.

## The current issue

The urban–rural dual division system has been a long-standing social issue and has been widely criticized in China (2). Xue and Li explored the impact of the integration of urban and rural medical insurance on migrant workers' overwork, based on panel data from 2016 and 2018. Xue and Li found that the integration of urban and rural medical insurance can significantly alleviate the excessive labor of migrant workers, and this alleviation especially affects work for migrant workers belonging to the older generation, from the central and western regions, working in the secondary industry and having a high level of education. They further explored the possible underlying mechanism and indicated that the integration of urban and rural medical insurance will improve the social identity and health level of migrant workers, and then reduce the probability of migrant workers' overwork. Continuity is a crucial part of the health care of the internal migrant population under the aforementioned condition of dual urban-rural division (3). Zhang, Wu, et al. explored the potential mechanism of how people-centered integrated care (PCIC) in medical alliances promotes the continuity for migrants, from dimensions of continuity, accessibility, and comprehensiveness, and also explored the moderated mediating role of respect. Zhang, Wu, et al. found that coordination had a direct effect on continuity and had a mediating effect on continuity via comprehensiveness and accessibility. They also showed that respect has a positive moderating effect on the relationship between coordination and comprehensiveness.

The number of migrant older adults following children (MEFCs) has gradually increased along with the aging and urbanization of the population in recent decades in China. In this context, Di et al. investigated the mediating effect of family support on the relationship between acculturation and loneliness among MEFCs. Based on 656 MEFC, Di et al. found that acculturation of MEFC had a direct negative association with loneliness and a direct positive association with family support, while family support exerted a direct negative effect on loneliness. They further quantified that the mediating effect of family support accounted for 14.0% of the total association between acculturation and loneliness. Bao et al. qualitatively explored the factors affecting MEFC urban assimilation in China and presented the corresponding strategies to promote urban assimilation. Bao et al. concluded that the urban life of migrant older adults is mostly community-based and the fundamental institutional barrier is a significant factor that influences the ability of these migrant older adults to live a stable urban life. They emphasized that it is especially important for MEFC to reconstruct close neighborhood relationships and regain the humanity affection of the traditional acquaintance society, and suggested that the Chinese government needs to promote a nationwide unified pension and health insurance system, so that migrant older adults can enjoy the same benefits as local older adult residents in the “inflow” area.

Three articles focus on medical insurance or service. Yao et al. explored the pattern of health insurance use of 15,302 internal migrants in mainland China. They found that ~71% of internal migrants were enroled in a social health insurance programme outside of their residential location and of those hospitalized, ~73% were admitted to a hospital at their migration destination. They further indicated that internal migrants who had a local insurance fund aligned with residency [adjusted odds ratio (AOR) = 2.642, 95% CI = 2.108–3.310, *p* < 0.001] and were enroled in employment-based insurance (AOR = 1.761, 95% CI = 1.348–2.301, *p* < 0.001) were more likely to use insurance funds for hospital care and paid less out-of-pocket (β = −0.183 for local funds, *p* = 0.017; β = −0.171 for employment-based insurance, *p* = 0.005) than others. Meng et al. explored the association of medical services with population migration. They found that migrants with rural household registration or migrants not covered by the New Rural Cooperative Medical System were more prone to return to their hometowns for medical service reasons. They also showed that middle-aged and older people who migrated across provinces have the highest tendency to return to their hometowns due to medical services, and young people who migrated across and within provinces had the lowest propensity to return to their hometowns. Following the passage of the Affordable Care Act (ACA) and the subsequent 2012 Supreme Court decision, some states in the U.S. elected to offer Medicaid coverage to adults with incomes up to 138% of the Federal Poverty Level while others did not (4). Guo and Zou investigated whether post-ACA Medicaid coverage differences play a role in insurance coverage and interstate migration flow of low-educated non-citizens. They found that the 2014 Medicaid expansion was associated with statistically significant increases in insurance coverage rates among low-educated non-citizens, but there is little evidence supporting an increase in the in-migration rate or a decrease in out-migration rate in expansion states relative to that of non-expansion states.

Three papers put a special focus on minorities. Liu et al. established several prediction models to assess the risk of HIV infection among internal migrant men who have sex with men (IMMSM). Zhang, Chen, et al. assessed associations of migration with quality of life (QoL) among adults with rare diseases. Zhang, Chen, et al. found that among rural adults with rare diseases, migration had a positive direct effect on physical and environmental QoL, a positive indirect effect on physical and social QoL through increased individual income, and a negative indirect effect on environmental QoL via reduced tangible support. On the contrary, neither direct nor indirect associations of migration with quality of life were significant among the urban participants group. Asim et al. explored the maternity care experience of 120 Pakistani ethnic minorities, who had given birth in Hong Kong (HK). Asim et al. found that Pakistani women born in Hong Kong expressed relatively less satisfaction with care, especially during pregnancy and labor and delivery, compared to Pakistani women; women with fluent or conversational English-speaking ability also felt comparatively less satisfied particularly with intrapartum and postnatal care in hospital; and the level of education had a negative association with satisfaction with care during pregnancy.

Other topics include alcohol consumption and depression. Kurshed et al. investigated the alcohol consumption behaviors of the general and Roma populations of Hungary. They found that Roma people experienced more alcohol-related harm than non-Roma and that gender and marital status differences act more intensely among Roma than non-Roma when considering alcohol-related harm. Xiong and Hu investigated the mediating role of self-esteem and the moderating role of belief in a just world between relative deprivation and depressive symptoms among 1076 rural-to-urban migrant children. Xiong and Hu identified a significant positive association between relative deprivation and depressive symptoms, and self-esteem partially mediates this association. They also found that belief in a just world moderated the association between relative deprivation and self-esteem.

Finally, there is an article on an interesting topic. Based on 129,803 observations from China, Zheng et al. investigated the association between migration distance and happiness, examined the heterogeneity of this association between urban and rural areas, and explored the transmission mechanism of migration distance on happiness by mediating the role of social integration. The researchers found that the distance from internal migration in China has a significant negative association with happiness, and this association is stronger among urban migrants. Social integration was also shown to be the potential mechanism through which migration distance works in happiness.

## Conclusions

Timely, reliable, and impactful evidence on migration and health will help guide policymakers in devising evidence-based policies and action plans to tackle migration aspects of the SDGs. This Research Topic highlights recent literature on the institutional barrier (urban-rural dual division system), migrant older adults following children, medical insurance or service of migrants and migrant minorities, and the health risk behaviors and well-being accompanied by migration.

## Author contributions

All authors listed have made a substantial, direct, and intellectual contribution to the work and approved it for publication.
